# Comparison of Ultrasound-Guided Erector Spinae Plane Block and Oblique Subcostal Transversus Abdominis Plane Block for Postoperative Analgesia After Laparoscopic Cholecystectomies: A Prospective Randomized Controlled Trial

**DOI:** 10.7759/cureus.93364

**Published:** 2025-09-27

**Authors:** Dharani M, Geetha Soundarya Udayakumar, Senthil Kumar Sivakumar, Raghuraman M Sethuraman, Vidhya Narayanan

**Affiliations:** 1 Anesthesiology, Sree Balaji Medical College and Hospital, Bharath Institute of Higher Education and Research (BIHER), Chennai, IND; 2 Orthopedics, Madras Medical College, Chennai, IND

**Keywords:** erector spinae plane (esp) block, laparoscopic cholecystectomy, oblique subcostal transverse abdominis plane, postoperative pain management, ultrasound-guided

## Abstract

Introduction

Laparoscopic cholecystectomy (LC) is a commonly performed minimally invasive surgical procedure that can result in significant postoperative discomfort. Pain following this surgery has both somatic and visceral components. Providing adequate pain relief after the procedure is essential to enable early ambulation, which is crucial for patient satisfaction and facilitating timely discharge. The application of ultrasound (US) in anesthetic practice has initiated a new era of US-guided blocks for postoperative analgesia, replacing traditional methods. This study was planned to evaluate the analgesic efficacy of US-guided erector spinae plane block (ESPB) and oblique subcostal transversus abdominis plane (OSTAP) block in patients undergoing elective LC, to compare the analgesic requirements in both groups, and to compare the numeric rating scale (NRS) scores in both groups.

Materials and methods

Sixty patients aged between 18 and 75 years, with American Society of Anesthesiology (ASA) Grades I and II, were enrolled in the study. They were randomized into two groups: one group that received a bilateral ESPB (Group E) at the T7 level and another that received a bilateral OSTAP block (Group O) with 20 mL of 0.375% bupivacaine, following intubation. The primary outcomes measured were the median NRS scores and the time to first analgesic requirement during the first 24 hours postoperatively. Secondary outcomes included total tramadol consumption, analgesic requirements, and complications related to the opioids or blocks.

Results

The ESPB group reported an NRS score < 4 up to 12 hours postoperatively. The NRS scores in Group E were significantly lower at 20 minutes, 40 minutes, 1 hour, 3 hours, 6 hours, and 12 hours post-surgery when compared to Group O (p < 0.05). The mean time to first rescue analgesic requirement was 12.2 ± 5.6 hours in Group E and 6.6 ± 2.84 hours in Group O, which was found to be statistically significant (p < 0.001). The total tramadol consumption per patient in 24 hours was significantly higher in Group O than in Group E (p < 0.001).

Conclusion

Bilateral US-guided ESPB provides effective analgesia, resulting in lower pain scores and reduced postoperative tramadol consumption within the first 12 hours for patients undergoing LC.

## Introduction

Cholecystectomy is the definitive treatment for symptomatic conditions of the gallbladder, such as cholecystitis and cholelithiasis. Laparoscopic cholecystectomy (LC) is the most commonly performed minimally invasive surgical procedure. However, despite its advantages, LC can result in significant postoperative discomfort. This discomfort is attributed to several factors, including the segmental innervation of pain pathways along the transabdominal fascial plane.

Specific sources of pain include somatic pain from the trocar entry incisions, visceral pain caused by peritoneal distention, and irritation of the diaphragm due to high intra-abdominal pressure and carbon dioxide (CO_2_) insufflation [1-3]. Some researchers suggest that visceral pain related to tissue trauma during gallbladder resection may be the most significant component of postoperative discomfort [4].

Effective postoperative pain management is essential for promoting early patient mobilization and speeding up recovery. A range of treatment options is available for postoperative pain relief. These include systemic opioids, non-opioid analgesics, and local anesthetic infiltration at the surgical site.

Regional anesthetic techniques, such as transversus abdominis plane (TAP) blocks, oblique subcostal TAP (OSTAP) blocks, paravertebral blocks, and erector spinae plane blocks (ESPBs), are crucial for effective pain management [5-8]. While opioids can cause side effects like nausea, vomiting, and itching, non-steroidal anti-inflammatory drugs (NSAIDs) may have negative effects on gastric, hepatic, and renal functions. On the other hand, peripheral nerve blocks can significantly reduce these systemic side effects.

In 2016, Forero et al. first described the ESPB for managing thoracic neuropathic pain [9]. This procedure involves injecting a local anesthetic between the transverse processes and the erector spinae muscles to perform the block [10]. Previous studies have indicated that this solution is likely to block the ventral rami, dorsal rami, and rami communicantes of the spinal nerves [9]. As a result, it can effectively alleviate both somatic and visceral pain. Various studies have theorized about the potential spread of local anesthetic drugs after injection into the erector spinae plane.

Chin et al. proposed that injecting 20 mL of anesthetic into the erector spinae plane would spread locally at least three segments up and four segments down [11]. To achieve optimal analgesic coverage, you must inject the drug at the vertebral level that corresponds to the midpoint of the desired field. Hannig et al. clearly established that injecting between the T7 and T9 levels effectively spreads the drug to the T6-T12 segments [12].

The subcostal approach to the TAP block (OSTAP) was described by Hebbard et al. as a method for providing postoperative analgesia, especially following upper abdominal surgeries [13]. Studies show that ultrasound (US)-guided OSTAP blocks can reduce postoperative pain and opioid use within the first 24 hours after LC.

The OSTAP block provides analgesia for somatic and parietal pain by blocking somatic pain fibers and the fibers of the thoracic intercostal nerves [14]. Visceral pain resulting from tissue damage during gallbladder removal is commonly considered the most significant factor following LC. It is known that TAP (transversus abdominis plane) and OSTAP blocks do not impact visceral nerves [10]. Therefore, an alternative strategy may be necessary to alleviate visceral pain as part of a multimodal analgesia approach.

## Materials and methods

This single-blind, randomized clinical study received Institutional Ethics Committee approval (Ref No. 002/SBMC/IHEC/2022/1695, dated March 18, 2022) and was registered with the Clinical Trials Registry of India (CTRI/2022/04/041909, dated April 19, 2022). It was conducted at Sree Balaji Medical College and Hospital from May 2022 to May 2024. All patients provided written informed consent for the block interventions and participation in the study. The study included patients aged between 18 and 70 years with an American Society of Anesthesiology (ASA) physical status of I or II, who were scheduled for elective LC surgery. The exclusion criteria for the study included individuals with coagulation disorders, infections at the injection site of the block, known allergies to local anesthetics, advanced liver or kidney failure, chronic opioid use, and a body mass index (BMI) of 35 kg/m² or higher.

In the operating room (OR), standard monitoring procedures included electrocardiography, non-invasive blood pressure measurements, end-tidal CO_2_ measurements, and assessments of peripheral oxygen saturation for all patients. Before the administration of anesthesia, baseline measurements of heart rate, as well as systolic, diastolic, and mean arterial pressures, were recorded. After placing a 22-gauge intravenous line, a 15 mL/kg isotonic saline infusion was initiated. All patients received premedication, which consisted of intravenous glycopyrrolate (0.2 mg), midazolam (1-2 mg), and antibiotic prophylaxis, following the hospital's protocol. The anesthesiologists in the OR performed anesthesia inductions using intravenous propofol (2-3 mg/kg), fentanyl (1 μg/kg), and atracurium (0.5 mg/kg) to facilitate endotracheal intubation. Anesthesia was administered using intravenous atracurium and sevoflurane, along with a gas mixture of oxygen and nitrous oxide. Patients were randomly assigned to two groups using a computerized randomization table by an uninvolved researcher.

The anesthesiologist retrieved a sealed envelope from a folder that indicated each randomized patient's treatment assignment. All regional blocks were performed by an anesthesiologist under US guidance who was not involved in anesthesia management, data collection, or analysis. The nurses in the Post Anesthesia Care Unit (PACU), as well as the anesthesiologists administering anesthesia in the OR and those collecting postoperative data, were unaware of the type of regional block performed. Depending on the group allocation, either a bilateral ESPB (Group E) or a bilateral OSTAP (Group O) block was carried out.

After intubation, patients in the first group (ESPB) were positioned laterally. A linear US probe (GE LOGIQ P7, GE Healthcare, Chicago, IL, USA) was placed at the T7 spinous process and then moved 3 cm laterally from the midline. The US landmarks were identified, including the T7 transverse process and the overlying erector spinae muscle. Under aseptic conditions, an 80 mm, 21-gauge block needle was inserted in-plane at an angle of 30-40° in a cranial-to-caudal direction until the tip made contact with the T7 transverse process. After performing hydro-dissection with 2-3 mL of isotonic saline solution to confirm the correct needle tip position, the anesthesiologist injected 20 mL of 0.375% bupivacaine and 4 mg of dexamethasone deep into the erector spinae muscle, noting the resulting lift of the muscle. This procedure was then repeated on the contralateral side with another 20 mL of 0.375% bupivacaine and 4 mg of dexamethasone solution.

In Group O, the patient was in a supine position. The anesthesiologist placed the US probe obliquely on the upper abdominal wall along the subcostal margin, moving it laterally to visualize the external oblique, internal oblique, and transversus abdominis muscles. The anesthesiologist positioned the needle to inject 20 mL of 0.375% bupivacaine and 4 mg of dexamethasone into the space between the rectus abdominis and transversus abdominis muscles, targeting the transversus abdominis fascia along the subcostal line. This procedure was then repeated on the opposite side with another 20 mL of the same bupivacaine and dexamethasone solution.

Fifteen minutes after the block, surgery commenced. At the end, all patients received ondansetron (4-8 mg) and intravenous paracetamol (1 g), followed by reversal with myopyrolate before extubation. Our primary objective was to measure the numeric rating scale (NRS) score during the first 24 hours postoperatively. The secondary outcomes included the timing of the first analgesic requests, total analgesic doses received in the 24 hours following surgery, and any complications that may arise.

The NRS is a numeric version of the visual analog scale (VAS) used to assess postoperative pain. Respondents indicate their pain intensity by selecting a whole number between 0 and 10. The scale ranges from 0 for "no pain" to 1 for "the worst pain imaginable." Changes in the NRS scores were recorded at specific intervals. These scores were documented without disclosing group assignments to the anesthesiologist during postoperative assessments conducted at 20 minutes, 40 minutes, 1 hour, 3 hours, 6 hours, 12 hours, 18 hours, and 24 hours. If a patient’s NRS score was 4 or higher, they were administered a tramadol injection (2 mg/kg) as rescue analgesia. If the NRS score remained above 4 one hour after the intravenous (i.v.) administration of tramadol, a second-line analgesic, diclofenac 75 mg i.v. infusion was given. Additionally, during the 24 hours, the time of administration of the first rescue analgesic was recorded. All patients in both groups received 1 g of i.v. paracetamol immediately after surgery, followed by a second dose 12 hours later. Since multimodal analgesia was employed, paracetamol was given every 12 hours instead of the usual eight-hour interval.

Postoperative vital signs were meticulously documented for each patient. Signs of local anesthetic toxicity were closely observed, as well as any indications of visceral or peritoneal injury. The occurrence of opioid-related side effects, including nausea and vomiting, was also recorded to ensure comprehensive postoperative care.

Sample size calculation

The sample size for the study was determined by referencing a previous research that indicated that a minimum of 30 patients per group was necessary to detect a 25% variation in the NRS score at the 120th minute postoperatively. This calculation was based on a desired power of 0.1 and a significance level of 95% (α = 0.05, β = 0.9).

The statistical analysis was conducted using IBM SPSS Statistics for Windows, Version 23.0 (Released 2013; IBM Corp., Armonk, NY, USA). To determine the significant differences between independent bivariate samples, we used the unpaired sample t-test. Additionally, the chi-square test was employed to assess significance in categorical data. In all statistical analyses, a probability value of less than 0.05 was considered statistically significant.

## Results

A total of 60 patients were enrolled in the study. The demographic data, ASA grading, and surgical duration were comparable between the two groups (Table 1).

**Table 1:** Descriptive variables of both groups Independent t-test and chi-square test were used to calculate the p-values. ASA: American Society of Anesthesiologists.

Parameters		Group E	Group O	p-value
Age (years) (mean ± SD)		47.2 ± 10.1	48.1 ± 11.2	0.7450
Sex	Male, n (%)	11 (36.7)	13 (43.3)	0.60
	Female, n (%)	19 (63.3)	17 (56.6)	
BMI (kg/m^2^) (mean ± SD)		29.2 ± 5.4	27.9 ± 4.9	0.3329
ASA	Grade I, n (%)	12 (40)	14 (46.6)	0.945
	Grade II, n (%)	18 (60)	16 (53.3)	
Duration of surgery (mean ± SD)		105.8 ± 15.7	110.2 ± 12.8	0.2390

**Figure 1:**
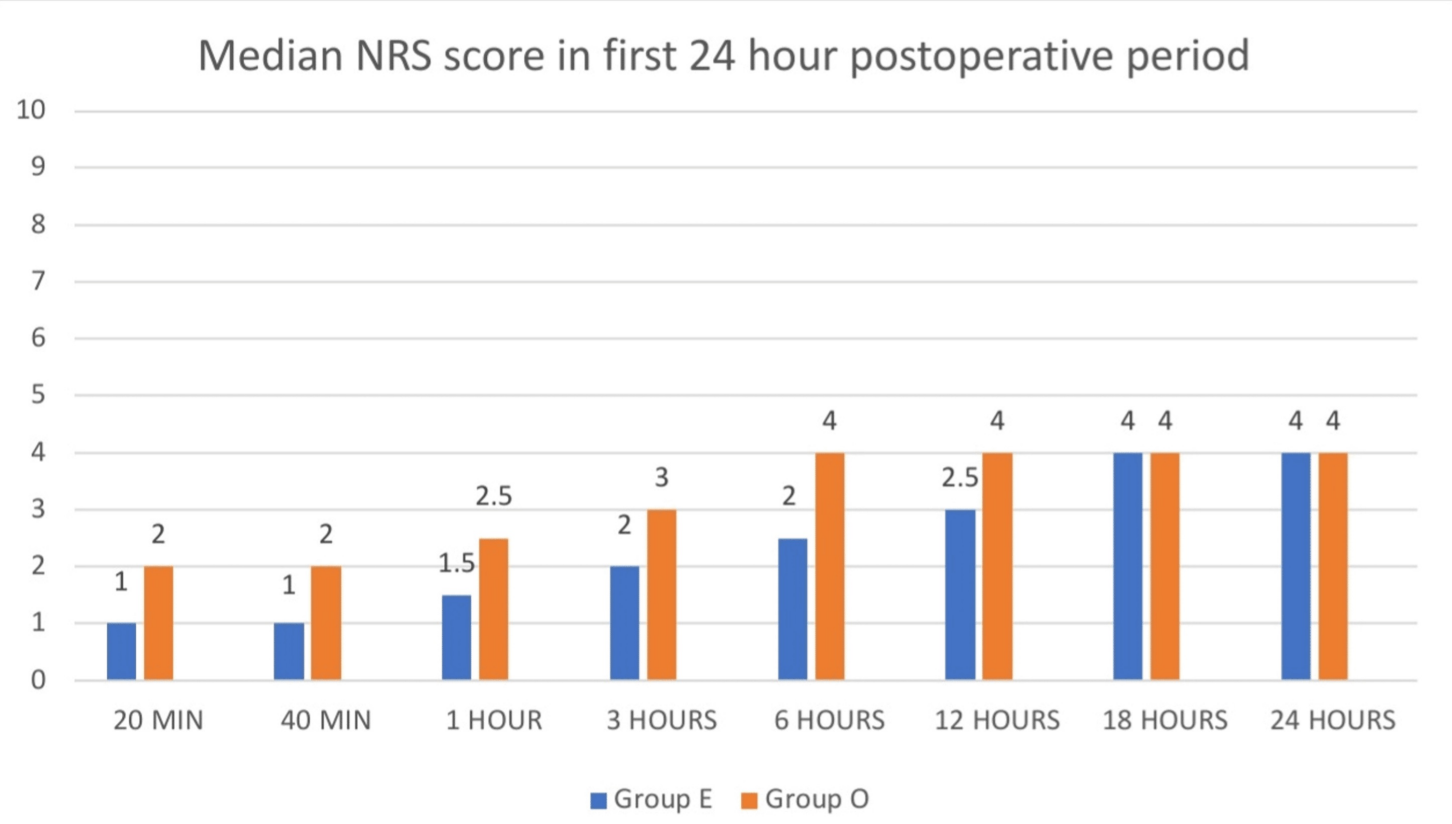
Median NRS scores in the first 24-hour postoperative period NRS: numeric rating scale.

The NRS scores were significantly lower at 20 minutes, 40 minutes, 1 hour, 3 hours, 6 hours, and 12 hours postoperatively in Group E compared to Group O (p < 0.05). The median NRS scores for all time periods are displayed in Figure 1.

NRS scores in Group E were significantly lower at postoperative intervals of 20 minutes, 40 minutes, 1 hour, 3 hours, 6 hours, and 12 hours compared to Group O (p < 0.05). However, there was no statistically significant difference between the groups at the 18-hour and 24-hour postoperative marks (p > 0.05) as shown in Table 2.

**Table 2:** Median NRS scores of both groups at different time points *Results are expressed as median (lower quartile and upper quartile). NRS: numeric rating scale.

Time duration	Group E*	Group O*	p-value
20 minutes	1 (0-2)	2 (1-2.75)	0.014
40 minutes	1 (1-2)	2 (1-3)	0.018
1 hour	1.5 (1-2)	2.5 (2-3)	0.021
3 hours	2 (1-3)	3 (2.5-4)	0.019
6 hours	2 (1.25-3)	4 (3-5)	<0.001
12 hours	2.5 (2-3)	4 (3.5-5)	<0.001
18 hours	4 (3.5-5)	4 (3-5)	0.892
24 hours	4 (3-5)	4 (3.5-5)	0.946

The average time to first required rescue analgesic was 12.2 ± 5.6 hours in Group E and 6.6 ± 2.84 hours in Group O, which was statistically significant (p < 0.001).

The total tramadol consumption per patient within 24 hours was significantly higher in Group O compared to Group E. Out of 60 patients, 22 required tramadol (7 from Group E and 15 from Group O). Additionally, 11 patients needed diclofenac as a rescue analgesic in the first 24 hours postoperatively (6 from Group E and 5 from Group O), as shown in Table 3.

**Table 3:** Postoperative analgesic requirement

Parameters	Group E	Group O	p-value
Total tramadol (mg) requirement in the first 24 hours, mean ± SD	135.1 ± 24.74	184.17 ± 28.04	<0.0001
Number of patients requiring tramadol in the first 24 hours, n (%)	07 (23.33 %)	15 (50%)	0.092
Number of patients requiring diclofenac in the first 24 hours, n (%)	06 (20%)	05 (16.66%)	0.333

There were no complications related to the block, such as hematoma, pneumothorax, or local anesthetic complications. A total of 10 patients experienced nausea (4 from Group E and 6 from Group O) and were treated with ondansetron. However, this finding was not statistically significant (p = 0.432).

## Discussion

In LC, postoperative pain has two primary components: somatic pain caused by trauma from the abdominal port and visceral pain resulting from gallbladder resection and pneumoperitoneum, which presents as a dull, aching sensation and may lead to referred pain in the right shoulder. Effective postoperative pain relief is essential for quality perioperative care. US-guided truncal blocks can enhance patient outcomes, reduce stress, improve satisfaction, decrease opioid usage, and lower the requirement for rescue analgesia [15].

This single-blind, randomized controlled study compared the analgesic efficacy of the ESPB with the OSTAP block in patients undergoing elective LC using 20 mL of 0.375% bupivacaine and 4 mg of dexamethasone. The results showed that postoperative NRS scores were significantly lower in Group E (ESPB) compared to Group O (OSTAP) for up to 12 hours after the procedure. Patients in Group E maintained NRS scores of less than 3 for up to 12 hours postoperatively, while Group O had scores below 3 only up to the third hour post-surgery. Additionally, Group E had a median NRS score of 2.5 maintained throughout the 12-hour postoperative period, whereas Group O exhibited a persistent median NRS score of 4 from the 6th hour until the 24th hour after the operation.

A study by Engineer et al. compared the efficacy of the ESPB and OSTAP block in LC [14]. They used 10 mL of 0.375% bupivacaine and 10 mL of 1.5% lignocaine with adrenaline. Results showed that the ESPB reduced postoperative pain scores to less than 3 for up to six hours, while the OSTAP group only maintained scores below 3 for the first hour. This suggests that administering the blocks before surgery could enhance analgesic effects [14].

Altıparmak B et al. also compared the efficacy of the ESPB and the OSTAP block in LC [16]. They used 20 mL of 0.375% bupivacaine for their blocks. Their findings showed that the NRS scores were significantly lower in the ESPB group compared to the OSTAP group at nearly all time points [16]. In the study conducted by Mounika et al., they found that the ESPB was more effective in terms of mean VAS scores over 24 hours compared to the OSTAP block [17].

Our study indicated that the mean time to first rescue analgesic requirement was 12.2 ± 5.6 hours in Group E and 6.6 ± 2.84 hours in Group O, which was statistically significant (p < 0.001). Our results were consistent with the findings of Engineer et al., who indicated that the time to first rescue analgesic was higher in the ESPB group compared to the OSTAP group [14].

Patients in Group E required significantly less tramadol overall (135.1 ± 24.74) than patients in Group O (184.17 ± 28.04), with a p-value of less than 0.0001. Furthermore, compared to patients in Group O, those in Group E requested analgesics less frequently. Nevertheless, it was discovered that the total diclofenac requirements of the two groups were similar. Out of 60 patients, 22 required tramadol (7 from Group E and 15 from Group O). Additionally, 11 patients needed diclofenac as a rescue analgesic in the first 24 hours postoperatively (6 from Group E and 5 from Group O).

Engineer et al. [14] found that fewer patients in the ESPB group needed rescue pain relief (tramadol) during the first 24 hours after surgery. In this group, 66% required rescue analgesics, compared to 88% in the control group (p = 0.019). Our results agree with Mounika et al. [17], indicating that the total opioid requirement in the first 24 hours was significantly lower in the ESPB group than in the OSTAP group (p = 0.034).

Verma et al. [18] reported that 66% of patients in the ESPB group required rescue analgesics (diclofenac) within the first 24 hours postoperatively, compared to 88% in the control group (p = 0.019). However, the total dose of diclofenac used was similar between the groups.

The addition of dexamethasone to both groups was found to provide a longer duration of analgesia and reduce opioid consumption in the postoperative period [19-21].

In this study, four patients from Group E and six from Group O experienced postoperative nausea and vomiting, but the difference was not statistically significant (p = 0.432). Mounika et al. [17] reported nine patients with nausea and vomiting (six from the ESPB group and three from the OSTAP group).

Similarly, Khalil et al. [22] found that the frequency of nausea and vomiting was greater in the OSTAP group compared to the ESPB group; however, the results were not statistically significant.

Our study acknowledges several limitations. First, we did not perform dermatomal mapping, which means we could not identify any potential patchy blockage or failure of the ESPB. Second, we administered the block while the patient was under anesthesia and did not wait to assess the effectiveness of the block before making the skin incision. Third, we did not observe or investigate all side effects related to opioids, such as respiratory depression, pruritus, and ileus. Additionally, we did not collect any outcomes related to patient experience or resource utilization. Finally, the study was limited by a small sample size and was conducted at a single center.

## Conclusions

Our study found that US-guided ESPB significantly reduces NRS pain scores and offers longer-lasting, higher-quality postoperative analgesia compared to the OSTAP block in patients undergoing LC. Additionally, this block reduces the consumption of postoperative analgesics. The study participants reported no adverse effects, and the findings were considered safe.
